# Electronic Health Records: A Gateway to AI-Driven Multimorbidity Solutions—A Comprehensive Systematic Review

**DOI:** 10.3390/jcm14103434

**Published:** 2025-05-14

**Authors:** Ignatios Ioakeim-Skoufa, Celeste Cebollada-Herrera, Concepción Marín-Bárcena, Vitor Roque, Fátima Roque, Kerry Atkins, Miguel Ángel Hernández-Rodríguez, Mercedes Aza-Pascual-Salcedo, Ana Fanlo-Villacampa, Helena Coelho, Carmen Lasala-Aza, Rubén Ledesma-Calvo, Antonio Gimeno-Miguel, Jorge Vicente-Romero

**Affiliations:** 1 Department of Drug Statistics, Division of Health Data and Digitalisation, Norwegian Institute of Public Health, 0213 Oslo, Norway; 2 Department of Pharmacology, Physiology and Legal and Forensic Medicine, Faculty of Medicine, University of Zaragoza, 50009 Zaragoza, Spain; 3 EpiChron Research Group on Chronic Diseases, Aragon Health Sciences Institute (IACS), Aragon Health Research Institute (IIS Aragón), Miguel Servet University Hospital, 50009 Zaragoza, Spain; 4 Drug Utilisation Work Group, Spanish Society of Family and Community Medicine (semFYC), 08009 Barcelona, Spain; 5 Research Network on Chronicity, Primary Care and Health Promotion (RICAPPS), Institute of Health Carlos III (ISCIII), 28029 Madrid, Spain; 6 Scientific Society for Biomedical Research, 07010 Palma de Mallorca, Spain; 7 Techn&Art—Technology, Restoration and Arts Enhancement Center, Polytechnic University of Guarda, 6300-559 Guarda, Portugal; 8 BRIDGES—Biotechnology Research, Innovation and Design for Health Products, Polytechnic University of Guarda, 6300-559 Guarda, Portugal; 9 Portuguese Society of Health Care Pharmacists (SPFCS), 3000-316 Coimbra, Portugal; 10 Drug Utilisation Section, Technology Assessment and Access Division, Australian Government Department of Health and Aged Care, Canberra, ACT 2606, Australia; 11 Support and Planning Unit, Directorate of the Canary Islands Health Service, 38006 Santa Cruz de Tenerife, Spain; 12 Primary Care Pharmacy Service Zaragoza III, Aragon Health Service (SALUD), 50017 Zaragoza, Spain; 13 Pharmacy Service, Virgen de la Victoria University Hospital, 29010 Malaga, Spain

**Keywords:** multimorbidity, artificial intelligence, electronic health records, data mining, comorbidity, machine learning, health information exchange, chronic disease, predictive analytics, disease management

## Abstract

**Background/Objectives:** Artificial intelligence (AI) plays an important role in real-world health research. It can address the complexities of chronic diseases and their associated negative outcomes. This systematic review aims to identify the applications of AI that utilize real-world health data for populations with multiple chronic conditions. **Methods**: A systematic search was performed in MEDLINE and EMBASE following PRISMA guidelines. Studies were included if they applied AI methods using data from electronic health records for patients with multimorbidity. **Results**: Forty-four studies met the inclusion criteria. The review revealed AI applications identifying disease clusters, predicting comorbidities, and estimating health outcomes such as mortality, adverse drug reactions, and hospital readmissions. Commonly used AI techniques included clustering methods, XGBoost, random forest, and neural networks. These methods helped identify risk factors, predict disease progression, and optimize treatment plans. **Conclusions**: This study emphasizes the increasing role of AI in understanding and managing multimorbidity. Integrating AI into healthcare systems can enhance resource allocation, improve care delivery efficiency, and support personalized treatment strategies. However, further research is needed to overcome existing limitations, particularly the lack of standardized performance metrics, which affects model comparability. Future research should adhere to commonly recommended evaluation practices to improve reproducibility and meta-analysis.

## 1. Introduction

Multimorbidity, defined as the presence of multiple chronic conditions in an individual, is the most common clinical presentation of chronicity in adults [1,2,3]. Over the past 20 years, its prevalence has risen dramatically, with the elderly being the most affected group [1]. While the likelihood of experiencing multimorbidity increases with age, the majority of individuals with multiple chronic conditions are actually under the age of 65 [1,4]. This trend suggests a broader shift in chronic disease patterns that affect people of all ages.

Multimorbidity can significantly deteriorate patients’ quality of life, and impact their physical, emotional, and social well-being, as well as that of their caregivers. It is linked to higher rates of healthcare utilization, often characterized by fragmented and uncoordinated care, along with an increased risk of polypharmacy, drug interactions, adverse events, and inappropriate prescribing [3,5,6,7,8,9,10,11]. Addressing these challenges requires a transition from disease-specific guidelines to more holistic, person-centered care models [12]. Effective prevention and management strategies could alleviate the burden on both individuals and public healthcare systems [4,13,14].

Policymakers face pressure to develop sustainable, evidence-based solutions that address the complex needs of chronic patients, especially those living with multimorbidity. There is an urgent need to design and implement effective strategies to prevent the onset and clinical evolution of multimorbidity by identifying high-risk individuals [1,15]. Despite some promising care models, robust evidence of their effectiveness remains limited [16].

Electronic health records (EHRs) provide a valuable source of real-world data to study patterns and risks associated with multimorbidity. Through various statistical methods, large-scale studies utilizing EHRs have characterized populations with multimorbidity, identified associated risks, and mapped multimorbidity patterns and trajectories over time [17,18,19,20,21,22]. Traditionally employed in epidemiological analyses, EHRs are now increasingly being examined through the lens of artificial intelligence (AI) and machine learning (ML). These technologies can analyze large datasets to classify diagnoses, uncover hidden associations, predict future complications, and support personalized screening strategies [23,24]. For example, various ongoing initiatives utilize data from the Clinical Practice Research Datalink (CPRD) to investigate the applications of AI in multimorbidity research [25]. One such initiative, the AIM-CISC program, leverages CPRD data to identify common combinations of long-term conditions and develop AI tools aimed at reducing adverse events [26]. Similarly, the CoMPuTE project applies AI techniques to identify individuals who are more likely to develop multimorbidity over time [27]. Despite this expanding body of research, there is a need to synthesize and evaluate how AI has been applied to EHRs in populations with multimorbidity. This review aims to fill that gap.

## 2. Materials and Methods

We conducted a systematic review of the peer-reviewed literature in MEDLINE and EMBASE, following the Preferred Reporting Items for Systematic reviews and Meta-Analyses (PRISMA) statement guidelines [28]. The detailed PRISMA checklist is provided in the Supplementary Material. The search strategy combined algorithms for multimorbidity, artificial intelligence, and electronic health records (see Appendix A).

Multimorbidity was defined as the presence of two or more chronic conditions, without limitation to specific diseases. This definition was consistently applied in both the search strategy and the screening process for inclusion and exclusion criteria. Studies were included if they involved the analysis of any combination of chronic diseases and utilized artificial intelligence methods within electronic health records.

**Figure 1:**
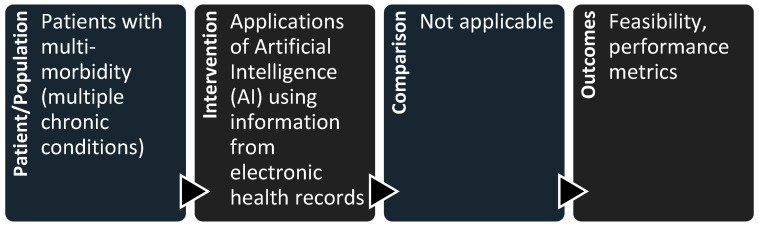
Application of the Patient/Population, Intervention, Comparison, and Outcomes (PICO) model to assess suitability of the identified articles for inclusion in the systematic review.

In this study, we included articles that met all the following criteria: (i) original articles; (ii) the full text is available; (iii) the paper is in English or Spanish; and (iv) it answers the research question. To properly address this last criterion, we applied the Patient/Population, Intervention, Comparison, and Outcomes (PICO) model [29], as shown in Figure 1.

We performed the literature review on 1 December 2024. Three researchers independently screened titles, abstracts, and full text when considered necessary, in pairs, following a double-blind method, to exclude irrelevant articles. Disagreements regarding study inclusion were resolved through structured discussion among all co-authors until consensus was reached. Reference lists of the included studies were also manually screened to identify additional eligible articles.

We collected data on several aspects, including the year of publication, country, study period, study type, follow-up duration, clinical setting, patient age, study population, disease classification, list of diseases, medications, drug classification, laboratory tests, social and lifestyle factors, health-related quality of life, study aims, sample size, outcomes, main findings, conclusions, limitations, and funding sources.

Additionally, we extracted variables related to AI, such as the AI tool used, the aim of the study, the type of data analyzed, whether supervised or unsupervised methods were employed, performance metrics (including accuracy, precision, recall, F1 score, and other relevant metrics), and details on missing data.

To evaluate the overall reliability of the evidence, we applied the Grading of Recommendations Assessment, Development, and Evaluation (GRADE) framework [30,31], which is effective for synthesizing findings from a wide range of observational studies. Considering the exploratory nature of this review and the significant differences in study designs, populations, data sources, and analytical methods, we determined that GRADE was an appropriate and sufficient approach to assess the strength of the existing evidence in this emerging field. A detailed report of this assessment can be found in the Supplementary Material, Table S1.

## 3. Results

### Literature Search Results

**Figure 2:**
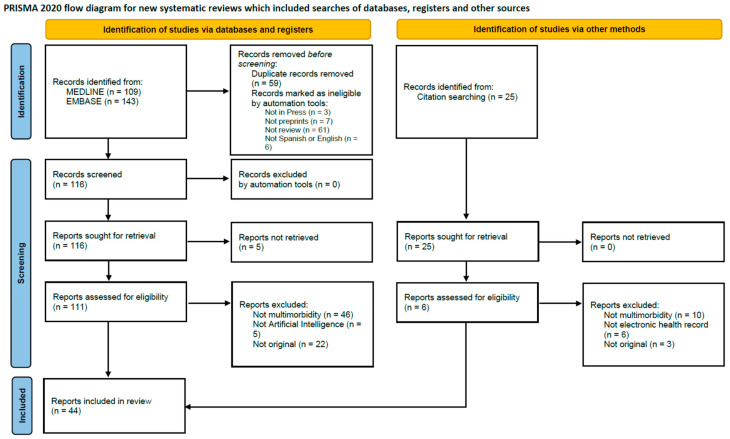
PRISMA 2020 flow diagram.

A total of 252 studies were identified through searches in MEDLINE (109 results) and EMBASE (143 results), as shown in the flow chart in Figure 2. In the initial stages, 59 instances of duplicate content were excluded, and 77 articles were identified as ineligible by automated tools. Of the remaining 116 publications, 5 were not retrieved for screening, resulting in 111 articles available for review. From these, 22 records were excluded because they were not original research, and 51 records were excluded for not addressing the topic of the study. This led to the inclusion of 38 articles in the review after the screening process. Additionally, by screening the titles of the references of the included studies, we identified 25 articles to be potentially relevant. Among these, six studies met the inclusion criteria and were included in the review. Before analysis, the dataset underwent a final deduplication procedure to eliminate any identical entries. Ultimately, the search process concluded with the inclusion of a total of 44 articles [3,17,32,33,34,35,36,37,38,39,40,41,42,43,44,45,46,47,48,49,50,51,52,53,54,55,56,57,58,59,60,61,62,63,64,65,66,67,68,69,70,71,72,73], the majority of which were retrospective observational studies.

Most of these studies employed AI techniques to cluster populations based on clinical characteristics, such as chronic diseases. They also aimed to identify clinical trajectories, tracking how multimorbidity clusters evolve over time, and to make predictions regarding future comorbidities, negative outcomes (including drug-related side effects and adverse reactions, as well as mortality), and health service utilization.

Table 1 summarizes general information about the included studies. Most of these studies aimed to identify individuals with similar clinical profiles, commonly referred to as multimorbidity clusters or networks [17,34,35,36,37,41,45,48,53,58,62,64,65,67,71]. They also explored different clinical trajectories [43,60,66], known as multimorbidity trajectories, examined the use of healthcare services [38,42,47], and predicted additional comorbidities or negative outcomes, including mortality [3,39,44,50,52,55,56,68,69,70], adverse drug reactions, and drug–drug interactions [40,61,62]. For a more detailed description of the included studies, please see the Supplementary Information. Common AI approaches applied in these studies included clustering methods (such as k-means or fuzzy c-means clustering) [34,48,58,62,63,64,65], Extreme Gradient Boosting (XGBoost) [3,37,39,69], random forest [3,39,40,56], and neural networks [3,17,35,44,52,55,57,68].

Out of the 44 studies included in the analysis, only 23 provided detailed performance metrics. Among these studies, there was significant variation in the metrics used to evaluate model performance. The most commonly reported metrics were precision, recall, accuracy, F1-score, and Area Under the Receiver-Operating Characteristic Curve (AUC). A summary of the range of reported values can be found in Table 2.

## 4. Discussion

This systematic review underlined applications of artificial intelligence in the study of multimorbidity. Research efforts focused on identifying individuals with similar clinical characteristics, often referred to as multimorbidity patterns, clusters, or networks. These studies also examined clinical paths and investigated patterns of healthcare utilization. Additionally, they aimed to predict the onset of further comorbidities or adverse outcomes, such as mortality, harmful drug reactions, and drug–drug interactions. Common analytical techniques included clustering, XGBoost, random forest, and neural networks.

The type of metrics used to assess performance varied significantly among studies. We observed that the most common metrics included precision, recall, accuracy, F1-score, and AUC. Due to methodological differences in the assessment of the performance of the AI models between studies, it was not possible to make any meaningful comparisons. This variability, along with differences in study design, disease focus, medication types, and clinical settings, further limited the comparability and generalizability of the findings. Another important observation was that many studies did not report detailed performance metrics, which made it difficult to assess validity and reliability. Transparent reporting that adheres to commonly recommended performance evaluation strategies is important to enhance reproducibility and facilitate plausible comparisons and meta-analyses.

### 4.1. Multimorbidity Patterns and Future Comorbidities

With cluster analyses, we can identify groups of patients with similar clinical profiles, also known as multimorbidity patterns. Common clustering techniques include K-means, hierarchical clustering, and fuzzy c-means. These methods help identify subtypes of multimorbidity by grouping patients with similar disease patterns, generating hypotheses and providing valuable insights into common disease pathways [34,48,58,62,63,64,65,74,75,76].

Advanced machine learning techniques, such as XGBoost, random forest, and neural networks, are widely used for predicting multimorbidity and other health outcomes. XGBoost, a gradient boosting algorithm, builds multiple weak learners in a sequential way, which make it effective for risk stratification and predicting the likelihood of patients developing additional chronic conditions. Its capability to manage imbalanced datasets is particularly useful for addressing rare multimorbidity patterns [3,37,39,69,74,75,76].

Random forest is an ensemble learning method that constructs multiple decision trees based on random subsets of data and averages their predictions. This approach reduces variance and overfitting while helping to identify key risk factors for multimorbidity. It classifies patients into multimorbidity clusters based on their disease profiles and highlights the most influential features, such as age, lifestyle, and specific diseases, in the progression of multimorbidity [3,39,40,56,74,75,76].

Neural networks, especially deep learning models, are effective for analyzing high-dimensional and complex datasets. These networks consist of multiple layers of artificial neurons that process data hierarchically. Denoising autoencoders, a specific type of neural network, are used to extract meaningful features from noisy medical data, enhancing the interpretability of large datasets. Furthermore, neural networks are valuable for predicting disease progression and supporting feature reduction, transforming high-dimensional medical data into more understandable formats [3,17,35,44,52,55,57,68,74,75,76].

It is projected that predictive analytics using AI will be one of the major digital health technologies to have a significant impact over the next 20 years [24]. Our review identified studies employing AI to predict comorbidities, including cardiovascular events [44,56,69]. Research is ongoing in the development of mortality risk estimation models based on the clinical profile of patients [55,68]. The most common techniques employed in predictive analytics with AI include advanced regression models and machine learning algorithms. Examples of such techniques include neural networks, decision trees, random forests and gradient boosting. As predictive analytics is increasingly incorporated into electronic health records, its use will become more pervasive. These tools can be used by healthcare professionals in daily clinical practice and in health policy-making to improve and individualize screening programs, leading to better allocation of clinical resources [24].

Although these models are often used for similar tasks, they differ in interpretability, computational complexity, and suitability for different types of clinical data. Tree-based methods like random forest and XGBoost are generally more interpretable and robust for tabular data; they often perform well with limited data and missing values. In contrast, neural networks—especially deep architectures—tend to require more data and computational resources, but they excel at capturing complex, non-linear relationships and handling unstructured data, such as clinical notes or longitudinal sequences. Therefore, selecting an AI model should depend not only on predictive accuracy but also on contextual factors such as data characteristics, clinical applicability, and explainability.

### 4.2. Drug Utilization and Drug-Related Adverse Events

Multimorbidity patients pose a treatment challenge due to the simultaneous presence of multiple diseases with different therapeutic requirements. Drug recommendation systems are AI prescription support algorithms that learn from the diagnoses and prescriptions in patients’ electronic health records to recommend more appropriate treatments, according to patients’ needs and with fewer drug–drug interactions. The findings in this regard substantiate the efficacy of drug recommendation systems, which enhance the efficiency of clinical decision-making while preserving or optimizing the safety of prescriptions [49,59,63,72].

Polypharmacy, the simultaneous use of multiple medications, is a common finding among individuals with multimorbidity. Although definitions of polypharmacy can vary across studies [77,78], it is widely recognized that the use of multiple medications increases the risk of inappropriate prescriptions, therapeutic cascades, adverse events, drug–drug interactions, drug–disease interactions, low adherence to treatment, increased healthcare utilization, and even mortality [1,9,79,80,81]. The application of advanced technological tools for patients with multimorbidity has shown promising results in predicting adverse drug reactions, identifying drug interactions, flagging potentially inappropriate medications, and preventing other drug-related negative outcomes [40,61,62]. For instance, using random forest models, Fahmi A et al. (2023) were able to predict he risks of adverse drug reactions and emergency hospital admissions [40]. Their results suggested that these models could be useful in prioritizing medication reviews.

### 4.3. Use of Healthcare Services

The utilization of healthcare services by chronic patients with multimorbidity is an important challenge for public health systems worldwide but also for the patients and their caregivers. In this systematic review, we found reports of AI techniques that could facilitate the extraction of psychosocial factors and a subsequent demonstration of their association with an elevated risk of hospitalization and emergency room visits [38]. This example underscores the importance of studying the factors contributing to a higher use of healthcare services to properly design and implement targeted interventions [82,83,84,85,86].

Additionally, comorbidity profiles in patients with schizophrenia were associated with higher readmission rates and use of psychiatric services. This underscores the need for an integrated psychiatric care model. Such comprehensive models would improve the monitoring and management of comorbidities in this patient demographic [38,42,47].

### 4.4. Clinical Scores

Scores developed using ML tools are presented as tools with great potential, as they lack the limitations of traditional scores, such as the use of a single dataset and being restricted to specific demographic groups. Most risk scores used in clinical practice are disease-specific, a limited approach that does not fully capture the heterogeneity of patients with multiple chronic conditions that are interrelated in variable ways. In contrast, ML models, which are developed using large datasets from patient records, have the capacity to offer highly predictive risk scales that are customizable, intuitive, and generalizable [54,73].

### 4.5. Limitations and Strengths

This study has several limitations that are common in most systematic reviews. However, we conducted a comprehensive literature search with clearly defined terms that allowed us to capture most relevant studies and thus to minimize the omission of relevant studies. The choice of databases, specifically MEDLINE and EMBASE, may be seen as a limitation since they do not encompass all pertinent literature. Furthermore, including only articles in English and Spanish could introduce a language bias. There is also a concern regarding publication bias, which occurs when non-significant results are less likely to be published. A significant limitation of this work is related to the quality and restrictions of each of the studies included.

All the studies included in this review were observational, and as no clinical trials were identified, the overall level of evidence of the included studies was low according to the GRADE framework. This rating reflects the inherent limitations of non-experimental designs, such as potential confounding factors and biases. Nevertheless, these studies provide valuable insights into the application of artificial intelligence in the context of multimorbidity, based on real-world data. While the findings should be interpreted with caution, they are very useful in generating hypotheses, informing clinical and policy discussions, and highlighting areas that require future high-quality research.

We observed considerable variability among the studies included in our analysis, particularly regarding study design, the AI methods employed (such as machine learning, natural language processing, and deep learning), data sources, definitions of outcomes, target populations, and the reporting of performance metrics. This variability led us to decide against conducting a meta-analysis. Due to important methodological and clinical differences among the studies that made it impossible to achieve a meaningful quantitative synthesis, we concluded that a narrative synthesis approach would be more appropriate for summarizing the existing evidence and identifying research gaps.

Given the novelty and rapid growth of this field, this systematic review is a crucial step in synthesizing the current evidence base. As more high-quality and standardized research becomes available, an updated systematic review should be conducted in the near future to include emerging studies and perform a meta-analysis.

Conversely, the review’s strengths include the utilization of a standardized and rigorous methodology, in accordance with PRISMA guidelines, and a comprehensive literature search conducted with clearly defined terms in the largest databases covering most of the studies on the topic (MEDLINE and EMBASE). The establishment of explicit inclusion and exclusion criteria aimed to enhance the transparency of the selection process, thereby improving the validity and reproducibility of the results.

### 4.6. Clinical Applications and Future Perspectives

This study shows the increasing role of AI in predictive analytics within the health sciences, particularly in addressing multimorbidity. AI has shown promising results in predicting clinical profiles and outcomes for chronic patients. This comprehensive review highlights several key opportunities where AI can significantly enhance patient care, including the identification of multimorbidity patterns, risk assessment, and the exploration of relationships between clinical profiles, additional comorbidities, medication use, health outcomes, medical and surgical procedures, utilization of healthcare services, and socio-economic variables.

A number of studies featured in this review focus on the prediction of adverse events, such as cardiovascular complications, hypertension, and stroke. The use of AI algorithms allows us to design and implement timely preventive interventions to improve patient outcomes, manage critical cases more effectively, and offer proper and high-quality care, while optimizing resource allocation and reducing the burden on the healthcare system. The capacity of AI tools to analyze large datasets in real time can boost the precision and effectiveness of such interventions.

As AI becomes an important tool in clinical research that is based on large datasets with information validated and recorded by healthcare professionals, the potential for transforming clinical practice and healthcare policy is significant. Predictive analytics can help healthcare providers to efficiently allocate resources and improve care delivery. It can also support research in advancing personalized medicine, identifying more accurate, effective, and patient-centered healthcare solutions, something that is particularly important for individuals with multiple chronic diseases.

Further studies are needed to refine predictive models and study their validity and applicability across different healthcare settings and populations. It is important to evaluate the real-world impact of AI-driven tools on clinical outcomes, care coordination, and healthcare utilization in patients with multimorbidity through well-conducted interventional studies. With solid evidence, we can achieve a safe and effective integration of AI into healthcare systems.

## 5. Conclusions

This systematic review highlights the transformative potential of AI in the study and clinical management of multimorbidity. By using information from large datasets from EHRs, we can apply AI models to identify complex disease patterns, predict additional comorbidities, and improve risk assessments for patients with multiple chronic conditions. These advancements empower healthcare providers to intervene proactively, in order to reduce the risk of adverse events such as cardiovascular complications, mortality, and hospitalizations, ultimately leading to improved patient outcomes.

AI models, such as XGBoost, random forest, and neural networks, allow us to assess risks, improve care delivery, and facilitate decision-making. In addition, they can assist us in addressing inappropriate polypharmacy, by identifying potential adverse reactions, drug–drug interactions, and other drug-related negative outcomes.

The use of AI in healthcare can improve resource allocation, enhance care efficiency, and facilitate the development of personalized treatment strategies. As the field evolves, there is a need for future research aiming to improve the performance of the models and ensure they can be effectively applied across different clinical settings and populations. Future research should focus on addressing existing gaps and limitations, such as the need for more detailed and standardized reporting, and on exploring AI’s potential to optimize healthcare delivery in real-world contexts. Conducting pragmatic clinical trials and interventional studies will be crucial for evaluating the impact of AI-driven tools on clinical outcomes, care coordination, and resource utilization. By generating high-quality evidence of AI technologies’ effectiveness in improving patient outcomes, these studies will lay a solid foundation for the safe and efficient integration of AI into healthcare systems, particularly for patients dealing with multimorbidity.

## Figures and Tables

**Table 1:** Characteristics of the included studies in the systematic review.

Author, Year [Ref]	Country	Clinical Setting	Study Population	Medication Usage	Laboratory	Social/ Lifestyle	Quality of Life, Health-Related	Sample Size (n)	AI Approach ^a^	AI Objective	Grade Score
Ageno A et al., 2023 [32]	Spain	Primary care	Multimorbidity	Yes	No	No	No	320	ML algorithms	Predict risk factors	Low ⨁⨁◯◯
Bendayan R et al., 2022 [33]	England	Specialized care	Severe mental illness	Yes	Yes	Yes	No	17,500	NLP (MedCAT)	Extract physical health data	Low ⨁⨁◯◯
Bolt H et al., 2023 [34]	England	Hospital	Acute Kidney Injury	No	No	No	No	133,488	Clustering	Profile clusters of underlying comorbidities	Low ⨁⨁◯◯
Chushig-Muzo D et al., 2022 [35]	Spain	Hospital	General population	Yes	No	No	No	15,162	Denoising autoencoder	Profile progression of chronic patients	Low ⨁⨁◯◯
Cruz-Ávila HA et al., 2020 [36]	Mexico	Hospital	Cardiovascular diseases	No	No	No	No	34,099	CVC	Estimate molecular relationships behind multimorbidity	Low ⨁⨁◯◯
Dashtban A et al., 2023 [37]	UK	Primary care	Chronic Kidney Disease	Yes	Yes	Yes	No	350,067	XGBoost, NB, K-NN, DT	Profile clusters of chronic kidney disease	Low ⨁⨁◯◯
Dorr DA et al., 2022 [38]	USA	Hospital	Multimorbidity	No	No	Yes	No	76,479	LR	Predict health care utilization	Low ⨁⨁◯◯
Dworzynski P et al., 2020 [39]	Denmark	Hospital	Type 2 diabetes mellitus	Yes	No	No	No	203,517	RLR, LR, RF, XGBoost	Predict future onset of chronic disease comorbidities	Low ⨁⨁◯◯
Fahmi A et al., 2023 [40]	UK	Hospital	Polypharmacy	Yes	No	Yes	No	532,732	RF	Predict ADR risk	Low ⨁⨁◯◯
Fränti P et al., 2022 [41]	Finland	Primary care and hospital	General population	No	No	No	No	3,800,000	M-algorithm	Profile multimorbidity clusters	Low ⨁⨁◯◯
Han X et al., 2022 [42]	China	Hospital	Schizophrenia and related disorders	No	No	No	No	8252	Association Rule Mining (ARM)	Predict health care utilization	Low ⨁⨁◯◯
Hayward CJ et al., 2023 [43]	England	Hospital	General population	No	No	Yes	No	375,669	AI-powered process mining	Estimate disease trajectories	Low ⨁⨁◯◯
Hossain ME et al., 2021 [44]	Australia	General	Type 2 diabetes mellitus	No	No	No	No	344	LR, SVM, DT, RF, NB, K-NN	Predict comorbid risk	Low ⨁⨁◯◯
Josephson CB et al., 2023 [45]	UK	Primary care and hospital	Epilepsy	Yes	No	Yes	No	1,032,129	NLP (CALIBRE)	Profile clusters of premature mortality	Low ⨁⨁◯◯
Khader F et al., 2023 [46]	USA and Germany	Hospital	Admitted to ICU ^b^	No	Yes	No	No	81,558	MDL	Improve diagnostic performance	Low ⨁⨁◯◯
Kueper JK et al., 2022 [47]	Canada	Primary Care	General population	No	No	Yes	No	221,047	Primary-care decision support tool	Improve diagnostic decision	Low ⨁⨁◯◯
Lai FTT et al., 2021 [48]	China and Switzerland	Hospital	General population	No	No	No	No	20,000	Hierarchical clustering	Profile multimorbid inpatients	Low ⨁⨁◯◯
Li R et al., 2023 [49]	China	Hospital	General population	Yes	No	No	No	6350	PIMNet.	Improve the medication recommendation	Low ⨁⨁◯◯
Linden T et al., 2021 [50]	USA	General	Epilepsy	Yes	Yes	No	No	132,265	DeepLORI	Predict risk for common comorbidities	Low ⨁⨁◯◯
Lip GYH et al., 2022 [51]	USA	Hospital	General population	No	No	No	No	4,289,481	ANN	Identify the relationships among comorbidity and other variables	Low ⨁⨁◯◯
Lu H et al., 2022 [52]	Australia	General	Chronic patients	No	No	No	No	19,828	ANN	Predict the comorbid risk of chronic diseases and their comorbidities	Low ⨁⨁◯◯
Ma H et al., 2022 [53]	China	Hospital	Admitted to hospital	No	No	No	No	144,207	Data mining	Identify associations between diseases	Low ⨁⨁◯◯
Mahajan A et al., 2021 [54]	USA	General	General population	Yes	Yes	Yes	No	992,868	ML algorithms	Predict multimorbidity risk scores	Low ⨁⨁◯◯
Nielsen AB et al., 2019 [55]	Denmark	Hospital	Admitted to ICU ^b^	No	Yes	No	No	11,896	ANN	Improves mortality predictions	Low ⨁⨁◯◯
Nikolaou V et al., 2021 [56]	UK	Hospital	COPD ^c^ and cardiovascular comorbidity	Yes	No	No	No	6883	RF, DT, XGBoost, MLR	Predict cardiovascular comorbidities	Low ⨁⨁◯◯
Oh SH et al., 2021 [57]	South Korea	General	General population	No	No	No	No	Unclear	CNN-based model	Predict similarity in multiple diseases	Low ⨁⨁◯◯
Prior TS et al., 2021 [58]	Denmark	Hospital	Idiopathic pulmonary fibrosis	Yes	Yes	No	Yes	150	Self-organizing maps	Profile comorbidity clusters	Low ⨁⨁◯◯
Sae-Ang, A et al., 2022 [59]	Thailand	Primary care	Diabetes, hypertension, or cardiovascular disease	Yes	No	No	No	3925	LR, NN, RF, MLP	Improve the drug prescription and verification	Low ⨁⨁◯◯
Shi X et al., 2021 [60]	Belgium	Primary care	Multimorbidity	No	No	No	No	65,939	Markov chains and WARM, Weighted Association Rule Mining	Estimate chronic conditions relations	Low ⨁⨁◯◯
Siebenhuener K et al., 2017 [61]	Switzerland	Hospital	Multimorbidity	Yes	No	No	No	1039	ML algorithms	Estimate combinations of chronic diseases and medications	Low ⨁⨁◯◯
Stafford G et al., 2021 [62]	Spain	Primary care	General population	Yes	No	Yes	No	916,619	Clustering	Profile multimorbidity and polypharmacy clusters	Low ⨁⨁◯◯
Strauss MJ et al., 2021 [17]	Austria	Hospital	General population	No	No	No	No	478,575	ANN	Identify disease phenotypes	Low ⨁⨁◯◯
Sun M et al., 2023 [63]	China	Hospital	Admitted to ICU ^b^	Yes	No	No	No	6350	Hierarchical clustering	Improve the medication recommendation	Low ⨁⨁◯◯
Uddin S et al., 2022 [3]	Australia	General	Chronic patients	No	No	No	No	29,100	LR, K-NN, NB, RF, XGBoost, MLP, CNN	Predict disease comorbidity and multimorbidity	Low ⨁⨁◯◯
Verhoeff M et al., 2023 [64]	Netherlands	Hospital	Oncologic patients with multimorbidity	No	No	No	No	22,133	Fuzzy c-means clustering	Profile multimorbidity clusters	Low ⨁⨁◯◯
Violan C et al., 2019 [65]	Spain	Primary care	General population	Yes	No	Yes	No	916,619	Fuzzy c-means clustering	Profile multimorbidity clusters	Low ⨁⨁◯◯
Wang T et al., 2022 [66]	UK	Hospital	Severe mental illness	No	No	No	No	7728	Temporal bipartite network model	Estimate hospitalization and multimorbidity profiles	Low ⨁⨁◯◯
Wesołowski S et al., 2022 [67]	USA	Hospital	Mother–child pairs	Yes	No	Yes	No	1,659,372	Poisson Binomial-based Comorbidity	Predict cardiovascular outcomes	Low ⨁⨁◯◯
Yang F et al., 2022 [68]	China	Hospital	Admitted to ICU ^b^	No	No	No	No	7491	RNN, MTL, LSTM-NN, RETAIN, Deepcare, DeepMPM-w/o-β, DeepMPM	Predict mortality risk	Low ⨁⨁◯◯
Ye C et al., 2018 [69]	USA	Primary care and hospital	General population	Yes	Yes	Yes	No	1,504,437	XGBoost	Predict hypertension risk	Low ⨁⨁◯◯
Zhang Y et al., 2015 [70]	USA	Hospital	Chronic kidney disease patients with multimorbidity	Yes	Yes	No	No	664	ML algorithm	Predict future state	Low ⨁⨁◯◯
Zhao B et al., 2023 [71]	USA	Hospital	Admitted to CCU ^d^	No	No	No	No	46,511	Graphical modeling	Improve diagnostic decision	Low ⨁⨁◯◯
Zheng H et al., 2021 [72]	USA	Primary care	Type 2 diabetes mellitus	Yes	Yes	Yes	No	16,665	Reinforcement learning	Improve health outcomes	Low ⨁⨁◯◯
Zulman DM et al., 2015 [73]	USA	Primary Care and Hospital	Hypertension	Yes	Yes	No	No	5997	Decision Support Systems (ATHENA—HTN)	Identify comorbidity interrelatedness	Low ⨁⨁◯◯

^a^ ANN (Artificial Neural Network), CNN (convolutional neural network), CVC (Comorbidity Network), DeepLORI (Deep personalized LOngitudinal convolutional RIsk model), DeepMPM (mortality risk prediction model based on deep learning), DT (decision tree), K-NN (K-nearest neighbor), LR (logistic regression), LSTM-NN (Long Short-Term Memory Neural Network), MDL (Multimodal Deep Learning), ML (machine learning), MLP (Multilayer Perceptron), MLR (multinomial logistic regression), MTL (multi-task learning), NB (Naïve Bayes), NLP (natural language processing), PIMNet (Patient Information Mining Network), RF (random forest), RLR (reference logistic regression), RNN (Recurrent Neural Network), SVM (Support Vector Machine). ^b^ ICU (Intensive Care Unit). ^c^ COPD (Chronic Obstructive Pulmonary Disease). ^d^ CCU (Critical Care Unit).

**Table 2:** Overview of performance metrics reported across included studies.

Study [Ref]	Model ^a^	Precision	Recall	Accuracy	F1 Score	Other ^b^
Bendayan R et al., 2022 [33]	NLP	0.82–1.00	0.85–1.00		0.91–1.00	
Cruz-Ávila HA et al., 2020 [36]	CVC					Jaccard: 0.121–0.828
Dashtban A et al., 2023 [37]	XGBoost			0.95	0.84–0.97	Se: 0.81–0.98
	NB			0.59–0.65	0.09–0.84	Se: 0.22–0.84
	K-NN			0.78	0.59–0.86	Se: 0.44–0.97
	DT			0.92	0.78–0.95	Se: 0.77–0.97
Dorr DA et al., 2022 [38]	LR					C-index: 0.53–0.81
Dworzynski P et al., 2020 [39]	RLR					AUC: 0.66–0.74
	LR					AUC: 0.68–0.77
	RF					AUC: 0.67–0.77
	XGBoost					AUC: 0.69–0.80
Fahmi A et al., 2023 [40]	RF					C-index: 0.62–0.66; DOR: 4.06–7.16
Hossain ME et al., 2021 [44]	LR	0.83	0.83	0.83	0.83	
	SVM	0.83	0.83	0.83	0.83	
	DT	0.84	0.92	0.83	0.88	
	RF	0.80	1.00	0.87	0.89	
	NB	0.82	0.75	0.79	0.78	
	K-NN	0.77	0.83	0.79	0.80	
Khader F et al., 2023 [46]	MDL					AUC: 0.70–0.77; Sp: 0.65–0.72; Se: 0.66–0.70; PPV: 0.34–0.40
Li R et al., 2023 [49]	PIMNet				0.69	PRAUC: 0.76; Jaccard: 0.54
Linden T et al., 2021 [50]	DeepLORI					Uno’s C-index: 0.72–0.77
Lip GYH et al., 2022 [51]	LR					C-index: 0.95
	ANN					C-index: 0.90
Lu H et al., 2022 [52]	ANN		0.70–0.90			AUC: 0.76–0.90; mAP: 0.47–0.68; NDCG: 0.54–0.74
Mahajan A et al., 2021 [54]	ML					AUC: 0.82–0.89; Sp: 0.75–0.83; Se: 0.72–0.82
Nielsen AB et al., 2019 [55]	ANN					AUC: 0.79; DOR: 0.41; PPV: 0.59
Nikolaou V et al., 2021 [56]	RF			0.86		Sp: 0.17–0.96; Se: 0.00–0.87; PPV: 0.00–0.98; NPV: 0.02–0.99
	DT			0.34		Sp: 0.14–0.97; Se: 0.06–0.88; PPV: 0.21–0.35; NPV: 0.69–0.79
	XGBoost			0.39		Sp: 0.15–0.97; Se: 0.06–0.89; PPV: 0.22–0.40; NPV: 0.70–0.84
	MLR			0.33		Sp: 0.15–0.97; Se: 0.05–0.90; PPV: 0.15–0.42; NPV: 0.74–0.86
Oh SH et al., 2021 [57]	CNN	0.52–0.85	0.61–0.89	0.54–0.86	0.55–0.92	
Sae-Ang A et al., 2022 [59]	NN	0.45	0.75			Hit: 0.97; NDCG: 0.77; Macro-AP: 0.25; Micro-AP: 0.62; Macro-AUC: 0.71; Micro-AUC: 0.88
	LR	0.45	0.75			Hit: 0.97; NDCG: 0.77; Macro-AP: 0.23; Micro-AP: 0.63; Macro-AUC: 0.69; Micro-AUC: 0.89
	RF	0.46	0.76			Hit: 0.97; NDCG: 0.76; Macro-AP: 0.33; Micro-AP: 0.64; Macro-AUC: 0.73; Micro-AUC: 0.89
	MLP	0.46	0.76			Hit: 0.97; NDCG: 0.79; Macro-AP: 0.32; Micro-AP: 0.67; Macro-AUC: 0.76; Micro-AUC: 0.90
Strauss MJ et al., 2021 [17]	ANN	0.00–1.00	0.00–1.00		0.04–0.78	
Sun M et al., 2023 [63]	HC				0.63	PRAUC: 0.71; Jaccard: 0.48
Uddin S et al., 2022 [3]	LR	0.75	0.74	0.74	0.73	
	K-NN	0.76	0.76	0.76	0.75	
	NB	0.61	0.63	0.63	0.54	
	RF	0.88	0.87	0.87	0.87	
	XGBoost	0.95	0.95	0.95	0.95	
	MLP	0.84	0.74	0.74	0.75	
	CNN	0.92	0.92	0.92	0.92	
Yang F et al., 2022 [68]	RNN	0.74	0.76		0.75	AUC: 0.83
	MTL	0.62	0.58		0.59	AUC: 0.64
	LSTM-NN	0.76	0.75		0.76	AUC: 0.83
	RETAIN	0.76	0.78		0.77	AUC: 0.83
	Deepcare	0.79	0.77		0.78	AUC: 0.79
	DeepMPM-w/o-β	0.77	0.78		0.77	AUC: 0.84
	DeepMPM	0.77	0.80		0.78	AUC: 0.85
Ye C et al., 2018 [69]	XGBoost					AUC: 0.87–0.92; Sp: 0.03–0.61; Se: 0.07–0.35; PPV: 0.01–0.51
Zhang Y et al., 2015 [70]	ML			0.07–0.75		FN: 0.00; FP: 0.00–0.25

^a^ ANN (Artificial Neural Network), CNN (convolutional neural network), CVC (Comorbidity Network), DeepLORI (Deep personalized LOngitudinal convolutional RIsk model), DeepMPM (mortality risk prediction model based on deep learning), DT (decision tree), HC (hierarchical clustering), K-NN (K-nearest neighbor), LR (logistic regression), LSTM-NN (Long Short-Term Memory Neural Network), MDL (Multimodal Deep Learning), ML (machine learning), MLP (Multilayer Perceptron), MLR (multinomial logistic regression), MTL (multi-task learning), NB (Naïve Bayes), NLP (natural language processing), PIMNet (Patient Information Mining Network), RF (random forest), RLR (reference logistic regression), RNN (Recurrent Neural Network), SVM (Support Vector Machine). ^b^ AUC (Area Under the Receiver-Operating Characteristic Curve), DOR (Diagnostic Odds Ratio), Hit (hit rate), FN (false negative), FP (false positive), Macro-AP (macro average precision), mAP (mean average precision), Micro-AP (micro average precision), NDCG (Normalized discounted cumulative gain), NPV (negative predictive value), PPV (positive predictive value), PRAUC (Area Under the Precision–Recall Curve), Se (Sensitivity), Sp (Specificity).

## Data Availability

Data are contained within the article and Supplementary Materials.

## Supplementary Materials

The following supporting information can be downloaded at: https://www.mdpi.com/article/10.3390/jcm14103434/s1, Table S1: A general overview of the studies included in the systematic review; File S1. PRISMA checklist: PRISMA 2020 main checklist and abstract checklist.

## Appendix A. Literature Search Strategy

The search strategy was a combination of three components: #1 (Multimorbidity) AND #2 (Artificial Intelligence) AND #3 (Electronic Health records). Below are the search algorithms for each component.

### Appendix A.1. Search Strategy in Medline

#### Appendix A.1.1. #1—Multimorbidity

(“multiple chronic conditions”[MeSH Terms] OR “multimorbidity”[MeSH Terms] OR “multiple chronic conditions”[Title/Abstract] OR “multimorbi*”[Title/Abstract] OR “multi morbi*”[Title/Abstract] OR “pluripatholog*”[Title/Abstract] OR “multidiseas*”[Title/Abstract] OR “multi diseas*”[Title/Abstract] OR “multiple conditions”[Title/Abstract] OR “multiple diseases”[Title/Abstract] OR “multiple health problems”[Title/Abstract] OR “multiple diagnos*”[Title/Abstract])

#### Appendix A.1.2. #2—Artificial Intelligence

(“Artificial Intelligence”[MeSH Terms] OR “Computer Heuristics”[MeSH Terms] OR “Expert Systems”[MeSH Terms] OR “Fuzzy Logic”[MeSH Terms] OR “Knowledge Bases”[MeSH Terms] OR “Machine Learning”[MeSH Terms] OR “Natural Language Processing”[MeSH Terms] OR “neural networks, computer”[MeSH Terms] OR “Robotics”[MeSH Terms] OR “Sentiment Analysis”[MeSH Terms] OR “Unsupervised Machine Learning”[MeSH Terms] OR “Supervised Machine Learning”[MeSH Terms] OR “Deep Learning”[MeSH Terms] OR “Data Mining”[MeSH Terms] OR “Support Vector Machine”[MeSH Terms] OR “Random Forest”[MeSH Terms] OR “Artificial Intelligence”[Title/Abstract] OR “Computer Heuristics”[Title/Abstract] OR “expert syst*”[Title/Abstract] OR “Fuzzy Logic”[Title/Abstract] OR “Knowledge Bases”[Title/Abstract] OR “Machine Learning”[Title/Abstract] OR “Natural Language Processing”[Title/Abstract] OR “neural networ*”[Title/Abstract] OR “Robotics”[Title/Abstract] OR “Sentiment Analysis”[Title/Abstract] OR “Machine Learning”[Title/Abstract] OR “Deep Learning”[Title/Abstract] OR “Data Mining”[Title/Abstract] OR “Support Vector Machine”[Title/Abstract] OR “Random Forest”[Title/Abstract] OR “cognitive analytics”[Title/Abstract] OR “cognitive syst*”[Title/Abstract] OR “general ai”[Title/Abstract] OR “strong ai”[Title/Abstract] OR “narrow ai”[Title/Abstract] OR “weak ai”[Title/Abstract] OR “input laye*”[Title/Abstract] OR “output laye*”[Title/Abstract] OR “hidden laye*”[Title/Abstract] OR “generative adversarial networ*”[Title/Abstract] OR “junction tre*”[Title/Abstract] OR “nearest neighb*”[Title/Abstract] OR “reinforced learning”[Title/Abstract])

#### Appendix A.1.3. #3—Electronic Health Records

(“electronic health records”[MeSH Terms] OR “health records, personal”[MeSH Terms] OR “medical records”[MeSH Terms] OR “electronic recor*”[Title/Abstract] OR “health recor*”[Title/Abstract] OR “medical recor*”[Title/Abstract] OR “patient recor*”[Title/Abstract] OR “personal health information”[Title/Abstract] OR “health diar*”[Title/Abstract] OR “medical transcrip*”[Title/Abstract] OR “health data”[Title/Abstract] OR “patient data”[Title/Abstract] OR “medical history”[Title/Abstract] OR “clinical history”[Title/Abstract])

### Appendix A.2. Search Strategy in Embase

#### Appendix A.2.1. #1—Multimorbidity

‘multiple chronic conditions’/de OR ‘multimorbidity’/de OR ‘multiple chronic conditions’:ab,ti OR ‘multimorbi*’:ab,ti OR ‘multi morbi*’:ab,ti OR ‘pluripatholog*’:ab,ti OR ‘multidiseas’:ab,ti OR ‘multi diseas*’:ab,ti OR ‘multiple conditions’:ab,ti OR ‘multiple diseases’:ab,ti OR ‘multiple health problems’:ab,ti OR ‘multiple diagnos’:ab,ti

#### Appendix A.2.2. #2—Artificial Intelligence

‘Artificial Intelligence’/de OR ‘Computer Heuristics’/de OR ‘Expert Systems’/de OR ‘Fuzzy Logic’/de OR ‘Knowledge Bases’/de OR ‘Machine Learning’/de OR ‘Natural Language Processing’/de OR ‘neural networks, computer’/de OR ‘Robotics’/de OR ‘Sentiment Analysis’/de OR ‘Unsupervised Machine Learning’/de OR ‘Supervised Machine Learning’/de OR ‘Deep Learning’/de OR ‘Data Mining’/de OR ‘Support Vector Machine’/de OR ‘Random Forest’/de OR ‘Artificial Intelligence’:ab,ti OR ‘Computer Heuristics’:ab,ti OR ‘expert syst*’:ab,ti OR ‘Fuzzy Logic’:ab,ti OR ‘Knowledge Bases’:ab,ti OR ‘Machine Learning’:ab,ti OR ‘Natural Language Processing’:ab,ti OR ‘neural networ*’:ab,ti OR ‘Robotics’:ab,ti OR ‘Sentiment Analysis’:ab,ti OR ‘Machine Learning’:ab,ti OR ‘Deep Learning’:ab,ti OR ‘Data Mining’:ab,ti OR ‘Support Vector Machine’:ab,ti OR ‘Random Forest’:ab,ti OR ‘cognitive analytics’:ab,ti OR ‘cognitive syst*’:ab,ti OR ‘general ai’:ab,ti OR ‘strong ai’:ab,ti OR ‘narrow ai’:ab,ti OR ‘weak ai’:ab,ti OR ‘input laye*’:ab,ti OR ‘output laye*’:ab,ti OR ‘hidden laye*’:ab,ti OR ‘generative adversarial networ*’:ab,ti OR ‘junction tre*’:ab,ti OR ‘nearest neighb*’:ab,ti OR ‘reinforced learning’:ab,ti

#### Appendix A.2.3. #3—Electronic Health Records

‘electronic health records’/de OR ‘health records, personal’/de OR ‘medical records’/de OR ‘electronic recor*’:ab,ti OR ‘health recor*’:ab,ti OR ‘medical recor*’:ab,ti OR ‘patient recor*’:ab,ti OR ‘personal health information’:ab,ti OR ‘health diar*’:ab,ti OR ‘medical transcrip*’:ab,ti OR ‘health data’:ab,ti OR ‘patient data’:ab,ti OR ‘medical history’:ab,ti OR ‘clinical history’:ab,ti
